# Discrete multi-physics: A mesh-free model of blood flow in flexible biological valve including solid aggregate formation

**DOI:** 10.1371/journal.pone.0174795

**Published:** 2017-04-06

**Authors:** Mostapha Ariane, Mohamed Hatem Allouche, Marco Bussone, Fausto Giacosa, Frédéric Bernard, Mostafa Barigou, Alessio Alexiadis

**Affiliations:** 1 School of Chemical Engineering, University of Birmingham, Birmingham, United Kingdom; 2 INSA Euro-Méditerranée, Université Euro-Méditerranéenne de Fès, Fez, Morocco; 3 LivaNova-Sorin, Saluggia, Vercelli, Italy; 4 Laboratoire Interdisciplinaire Carnot de Bourgogne, Université de Bourgogne Franche-Comté, Dijon, France; Tianjin University, CHINA

## Abstract

We propose a mesh-free and discrete (particle-based) multi-physics approach for modelling the hydrodynamics in flexible biological valves. In the first part of this study, the method is successfully validated against both traditional modelling techniques and experimental data. In the second part, it is further developed to account for the formation of solid aggregates in the flow and at the membrane surface. Simulations of various types of aggregates highlight the main benefits of discrete multi-physics and indicate the potential of this approach for coupling the hydrodynamics with phenomena such as clotting and calcification in biological valves.

## Introduction

Computational fluid dynamic (CFD) simulations of biological valves have steadily improved over the years; however, procedures accounting for the formation of actual solid aggregates, such as calcifications or clots, have not been implemented yet. At the same time, researchers have also devised mathematical models for clot formation and growth; however, these models have been developed independently and are not usually associated to the dynamics of the valve. We propose a particle-based method that, by taking advantage of its mesh-free nature, can compute the fluid dynamics, together with valve deformation and formation of solid aggregates.

**Fig 1:**
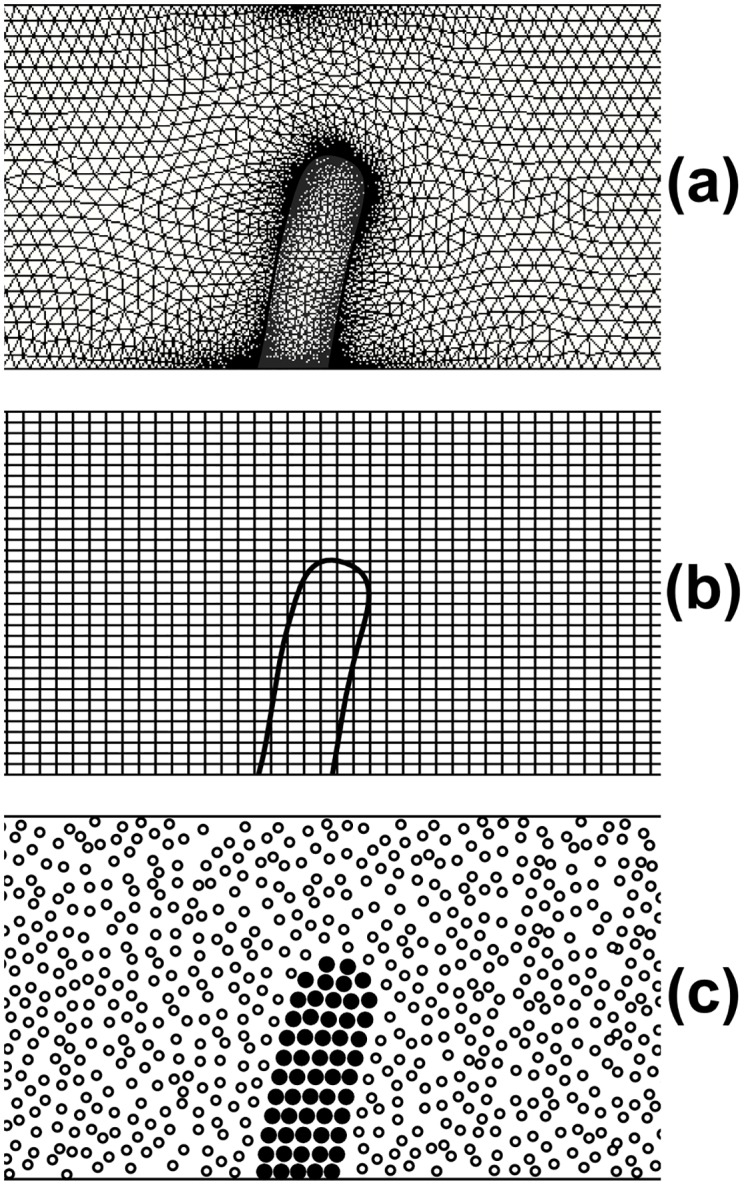
FSI algorithms: (a) conforming mesh, (b) non-conforming mesh, (c) mesh-free.

In general, the simulation of biological valves, where a solid structure interacts with the surrounding flow, constitutes a fluid-structure interaction (FSI) problem. The algorithms to solve FSI problems may be broadly classified into two categories [1]: conforming mesh methods (Fig 1a) and non-conforming mesh methods (Fig 1b). Conforming mesh methods [2] divide the computational domain in two parts: (i) a part occupied by the liquid where the Navier-Stokes equations are solved, and (ii) a part occupied by the structure where the stress-deformation equations are solved. Since the structure moves and/or deforms with time, re-meshing is needed as the solution advances. Non-conforming mesh methods, most notably the Immersed boundary methods (IBM) [3], treat the interface between the fluid and the structure as a constraint and the force exerted by the structure to the fluid becomes a source term in the momentum equation. As a result, the fluid and solid equations are solved independently and re-meshing is not necessary. Both methods, however, have difficulties handling phenomena such as calcification and clotting that involve some sort of transition where part of the liquid transforms into a solid. In general, attempts to account for the formation of solid aggregates in CFD/FSI studies are based on ‘numerical artifices’ such as fluids with higher viscosities to mimic clotting [4], or membranes with higher stiffness to mimic calcification [5].

For a different approach see [6–9].

On the other hand, modelling of clot formation and growth had followed an independent path that, in some cases, has brought to particle-based techniques such as Lattice-Boltzmann [10] or Coarse grained Molecular Dynamics [11]. In general, however, these models assume simple hydrodynamic conditions and/or refer to straight blood vessels with not moving/deforming parts. Additionally, coupling fluid dynamics and solid deformation can be implemented with the Smoothed Particle Hydrodynamics method [12, 13]. But, it can’t easily account for other phenomena such as contact mechanics and agglomeration.

In order to account for the fluid dynamics, the valve deformation, and the formation of solid aggregates at the same time, we propose *discrete multi-physics*: a mesh-free approach, where computational particles are employed for both the flow and the structure (Fig 1c). With this method, the distinction between liquid and solid depends exclusively on the types of forces that act on each particle: pressure and viscous forces characterize liquid particles, while elastic forces characterize solid particles. By changing the type of forces on specific groups of particles, we can change their status form liquid to solid and vice versa. In [14], we used this idea to model melting and solidifying flows; in this paper, we extend it to the formation of agglomerates in biological valves.

This article is organized as follows. Initially, we discuss the basic ideas behind our discrete multi-physics technique and describe the geometry used in the simulations. Next, we validate the model against both traditional modelling techniques and experimental data. Finally, we introduce the formation of solid aggregates at the membrane surface and in the flow.

The objective of this paper is to apply discrete multi-physics to biological valves in general. For this reason, we do not focus on a specific type of valve at this stage. However, in order to test our model in the most challenging scenario, we chose dimensions and velocities similar to those occurring in aortic valves. We consider these conditions to be the most challenging scenario because (i) they involve higher velocities, which generate complex recirculation patterns (see section Results), and (ii) they involve higher stresses, which generate larger membrane deformations (see section Results). From this point of view, the fact that we simulate bicuspid valves, while the aortic valve has three leaflets, is not a limitation. Given the same mechanical stress, in fact, deformations are higher in bicuspid valves than in tricuspid valves [15]. Therefore, by forcing velocities that are typical of aortic valves in bicuspid valves, we test our model under conditions that are even more critical (i.e. produce higher deformations) than those occurring in aortic valves.

## Modelling

### Discrete multiphysics

Our discrete multi-physics approach is based on the so-called discrete multi-hybrid system (DMHS). This technique combines various mathematical models to achieve a representation of fluid-structure interactions and solid-liquid systems that is not attainable with each model separately. Elsewhere [16], we showed that the linkage of different models is mathematically complex and computationally time consuming. In order to facilitate this, the DMHS combines models that share a common discrete (particle-based) paradigm, such as SPH (Smoothed Particle Hydrodynamics), CGMD (Coarse-grained Molecular Dynamics), DEM (Discrete Element Method) or BD (Brownian Dynamics).

In this study, models for solid contact/collision (i.e. DEM), or for fluctuating hydrodynamics (i.e. BD) are not necessary; consequently, the coupling is limited to SPH (liquid phase) and CGMD (solid phase). For the numerical solution, the model was implemented in LAMMPS [17]. A mathematical introduction to SPH and CGMD and the approach used to couple the models is given in S1 Appendix. Specific details of the DMHS and other mesh-free hybrid techniques can be found in [14, 18–21]. In previous DMHS publications, there is an interchangeable use of the terms CGMD and Mass-Spring Model (MSM). This depends on the fact that these articles cover different scales. Articles dealing with microscopic scales use CGMD, whereas articles dealing with macroscopic scales use MSM. Mathematically, however, the two techniques are equivalent. In the main text, we prefer MSM, which is more consistent with the scale under investigation. In S1 Appendix, we use CGMD, which is more consistent with the original DMHS formulation.

### Geometry

**Fig 2:**
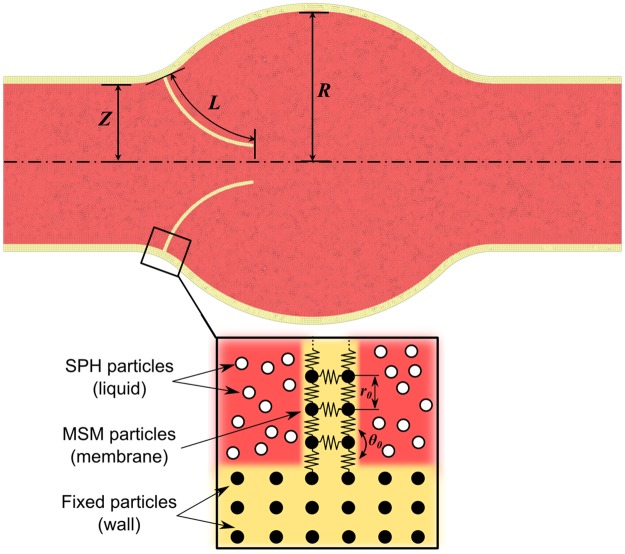
Valve geometry and types of particles used in the simulations.

We use a 2D simplified geometry for modelling a generic bicuspid valve as illustrated in Fig 2. The channel half-thickness is *Z* = 0.0125 m, the length of the membrane is *L* = 0.016m and the radius of the circular area is *R* = 0.0215 m.

As mentioned above, the DMHS combines various particle-based modelling techniques. In this study, the simulations are based on two models and three types of particles: SPH particles for the fluid, fixed SPH particles for the walls and MSM particles for the flexible leaflets (membrane). Periodic boundary conditions are used at the inlet/outlet. To model the Young modulus *E* and the flexural rigidity *F* of the membrane, the MSM particles are joined together by numerical ‘springs’ and ‘hinges’, as described in S1 Appendix. The relation between the spring (*k*_*b*_) and hinge (*k*_*a*_) constants and the actual Young modulus and the flexural rigidity is given in S2 Appendix.

### Pulsatile flow

In order to test the model in the most critical conditions, we target high velocities typical of cardiac valves such as the aortic valve. The flow is pulsatile and corresponds to a normal cardiac output of 5.5 L min^-1^, a beat rate of 72 bpm and an aortic pressure of 100 mmHg. This frequency gives a peak velocity of around 0.9 m s^-1^. In the simulation, we force the flow by means of a sinusoidal pressure *P* gradient

$$\frac{dP}{dx}=A\text{sin}(\frac{2\pi }{T}t),$$ (1)

where *A* is the force amplitude and *T* the period. To obtain this pressure gradient in the simulations, we impose to each liquid particle the acceleration

$$g=g_{0}\text{sin}(2\pi ft),$$ (2)

with *g*_*0*_ = 500 m s^-2^ and oscillation frequency *f* = 1/*T* (*T* = 1 s). Under these conditions, we reach high velocities but the flow remains laminar. We focus on the laminar regime for two reasons: (i) blood flow under normal conditions is laminar, and (ii) we want to test, at this stage, the accuracy of our model without dealing with the additional complexity of turbulence.

### Dimensionless analysis

In Section *Membrane deformation*, we compare our simulations with experimental data. The comparison is based on specific dimensional groups that are defined in this section.

Dimensional analysis bring to three fundamental groups Re (Reynolds Number), N_f_ (dimensionless frequency) and Λ (geometric ratio), defined as

$$\text{Re}=\frac{\rho UZ}{\mu },$$ (3)

$$N_{\text{f}}=\frac{\rho f^{2}d^{5}}{F/L},$$ (4)

and

$$\Lambda =\frac{Z}{L},$$ (5)

where *ρ* is the density of the fluid, *U* is a reference velocity (here we use the max velocity in the channel), *Z* the half-thickness of the channel, *μ* the fluid viscosity, *f* the oscillation frequency, *d* the membrane thickness, *F* the flexural rigidity, and *L* the length of the membrane. The computational particles used in our simulations are point particles; strictly speaking, they do not have an actual thickness. Their thickness is the result of the repulsive forces acting on the particles to impose no-penetration boundary conditions (see S1 Appendix). The value of *d* in eq 4, therefore, is calculated from *k*_*a*_ and *k*_*b*_ as discussed in S2 Appendix. The value of the Young modulus *E* does not compare explicitly in any of the dimensionless numbers above; this is due to the fact that *F* and *E* are interchangeable as discussed in S2 Appendix. In theory, we should also account for another dimensionless group based on *R* (radius of the convex area in Fig 2). In practice, however, this group is not necessary as discussed in Section *Membrane deformation*.

Each dimensionless number provides specific information about the geometric constants and the physical forces acting in the system. Re indicates the extent of the inertial forces with respect to the viscous forces. N_f_ indicates the membrane resistance with respect to the stress generated by the oscillating flow. Λ indicates the geometric ratio between the channel thickness and the membrane length. In comparing the simulations with the experimental data (see Section *Membrane deformation*), we found that the group

$${\text{N}}_{\text{R}}=\text{Re}.{\text{N}}_{\text{f}}=\frac{\rho^{2}f^{2}d^{5}UZ}{\mu F/L}$$ (6)

is particular relevant. This dimensionless number compares the effect of the forces that tend to deform the membrane (numerator) with those that tend to oppose the deformation (denominator). In Section *Membrane deformation*, we show that different geometries and flow conditions generate the same type of membrane deformation if N_R_ is the same.

## Results

There are two types of parameters required for the simulations: model parameters and simulation parameters. The first group consists of internal parameters used by the SPH and MSM solvers; the second refers to the operative conditions. This Section focuses on the second group (i.e. *Z*, *L*, *R*, μ, *ρ* and *F*); the internal parameters (e.g. *k*_*a*_, *k*_*b*_, number of particles, time step, smoothing length, etc.) can be found in S3 Appendix.

The geometric parameters *L*, *Z*, *R* are given in Section *Geometry* (see also Fig 2). All the simulations assume blood as liquid medium. Blood is a viscoelastic fluid, but in flow simulations [15, 22, 23] is often considered Newtonian. In our calculations, we also use the Newtonian approximation with *ρ* = 1056 kg m^-3^ and *μ* = 0.0035 Pa s. We consider membranes with different flexural rigidities, the specific value of *F*, for each case, is given in Section *Membrane deformation*.

This section is divided in three parts. The first part is dedicated to the flow, and we validate our results against traditional CFD simulations. The second part is dedicated to the membrane, and we validate our results against experimental data. The third part focus on the formation of solid aggregates, and highlights the main advantages of the DMHS in modelling biological valves.

### Hydrodynamics

We compare results obtained with our model with traditional CFD simulations performed with Abaqus 6.14^®^ with the same geometry and under similar flow conditions. In these simulations, the membrane is fixed in order to focus solely on the hydrodynamics. This is done on purpose: if at this stage we had considered both the fluid and the membrane together, we could not have distinguished whether potential errors originated from the fluid dynamics or the membrane mechanics.

**Fig 3:**
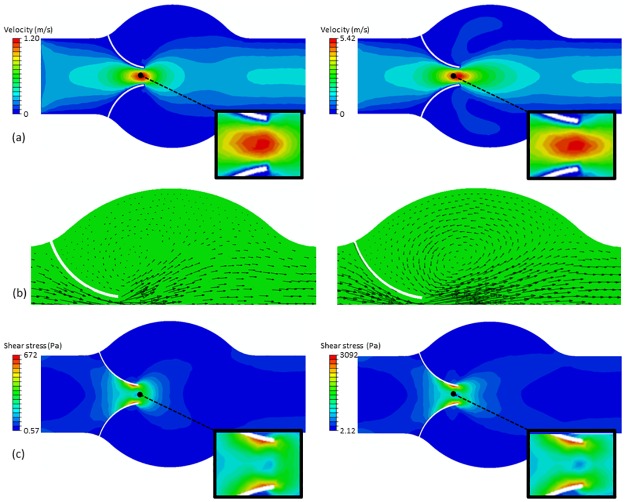
Velocity magnitude (a), velocity vectors (b) and shear stress (c) at 0.2 m s^-1^ (left) and 0.9 m s^-1^ (right) calculated with CFD.

**Fig 4:**
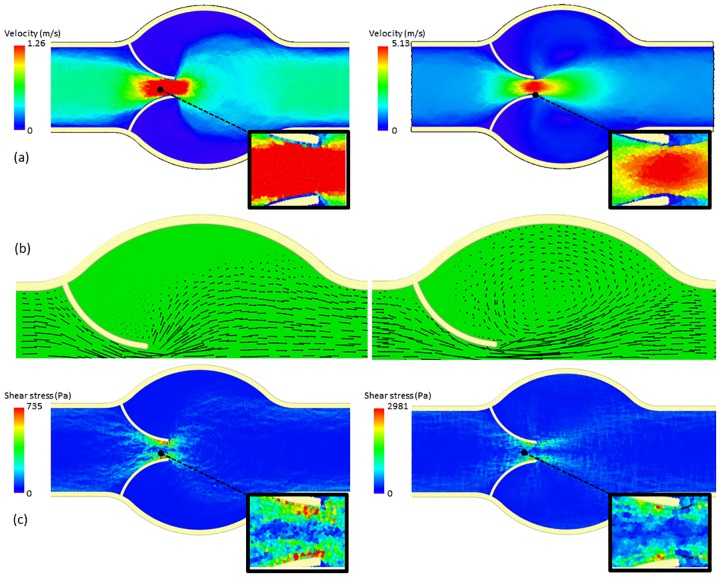
Velocity magnitude (a), velocity vectors (b) and shear stress (c) at 0.2 m s^-1^ (left) and 0.9 m s^-1^ (right) calculated with the DMHS.

Calculations are run at two constant inlet velocities, 0.2 m s^-1^ and 0.9 m s^-1^. Because of the different nature of the two modelling techniques, the inlet/outlet boundary conditions (b.c.) are not the same. The DMHS uses periodic inlet/outlet b.c., while in the CFD simulation the inlet has constant velocity and the outlet fixed pressure. Fig 3 shows the CFD results; Fig 4 shows the DMHS results.

Comparison between Figs 3 and 4 shows a good agreement between the CFD and the DMHS calculations. Both models, in particular, capture the recirculation zones in the circular chamber in the centre. For the velocity, minor differences (2–5%) can be found at the tip of the valve. These differences depend on the different inlet conditions between the DMHS and the CFD model and the nature of the discretization method (particles vs. mesh).

Another important variable often reported in literature is shear stress (Figs 3c and 4c). But also in this case CFD and DMHS results are similar. Both models, in particular, identify a region of high stress near, but not exactly at, the end of the leaflets.

### Membrane deformation

In this section, we account for the flexibility of the leaflets and calculate both the flow and the membrane dynamics. For validation purposes, we compare the membrane deformation observed during the simulations with those obtained experimentally by [24]. In both simulations and experiments the valve has two leaflets and the flow is pulsatile. The geometric conditions, however, are not exactly the same: in [24], in fact, *L* = 0.0263 m, *Z* = 0.015m, and the channel is straight without the circular chamber in the centre (this is why, in Section *Dimensionless analysis*, we did not introduce a forth dimensionless number). Additionally, our simulations are 2D and based on blood, while [24] employ water in a rectangular channel with depth *w* = 0.05 m. Table 1 gathers all the parameters used in the simulations and in the experiments. The values of *k*_*a*_ and *k*_*b*_ corresponding to a specific *F* in the simulations are given in S3 Appendix. The values of *d* for the simulations are calculated according to the procedure described in S2 Appendix.

**Table 1:** Geometric parameters, fluid conditions, and membrane constants used in the simulations and in the experiments.

	Simulations	Experiments	Membranes
*Z*	0.0125 m	0.015 m	All
*L*	0.016 m	0.026 m	
*ρ*	1056 kg m^-3^	1000 kg m^-3^	
*μ*	0.0035 Pa s	0.001 Pa s	
Λ	0.78	0.58	
*U*	0.9 m s^-2^	0.42 m s^-2^	Soft
*d*	0.0003 m	0.0004 m	
*f*	1 s^-1^	0.5 s^-1^	
*F*	0.008 kg m^-3^ s^-2^	2.30·10^−7^ kg m^-3^ s^-2^	
Re	3259	6300	
N_f_	6·10^−10^	3.11·10^−10^	
N_R_	1.96·10^−7^	1.96·10^−7^	
*U*	0.9 m s^-2^	0.51 m s^-2^	Intermediate
*d*	0.00016 m	0.0004 m	
*f*	1 s^-1^	0.167 s^-1^	
*F*	0.056 kg m^-3^ s^-2^	2.30 10^−7^ kg m^-3^ s^-2^	
Re	3259	7650	
N_f_	8.12·10^−11^	3.47·10^−10^	
N_R_	2.65·10^−7^	2.66·10^−7^	
*U*	0.9 m s^-2^	0.2 m s^-2^	Hard
*d*	0.0003 m	0.0004 m	
*f*	1 s^-1^	0.333 s^-1^	
*F*	0.12 kg m^-3^ s^-2^	4.61 10^−6^ kg m^-3^ s^-2^	
Re	3259	3000	
N_f_	6.36·10^−12^	6.9·10^−12^	
N_R_	2.07·10^−8^	2.07·10^−8^	

After running a large number of simulations and observing how the membrane deforms under various flow conditions, we realized that the fundamental group that affects the membrane dynamics is N_R_ (eq 6). Therefore, we chose specific values of *f*, *g*_0_ (which gives *U*), *k*_*a*_ and *k*_*b*_ (which give *F* and *d*) to obtain in our simulations the same N_R_ of the experiments.

**Fig 5:**
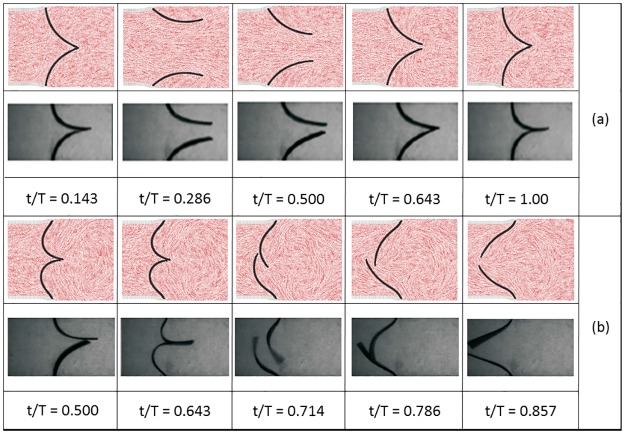
Comparison between simulations and experiments from [24]: (a) intermediate membrane, (b) soft membrane.

**Fig 6:**
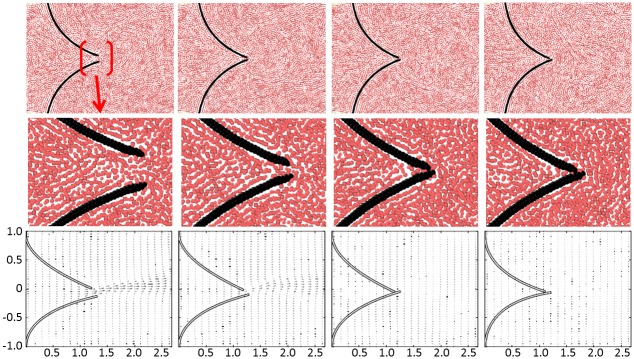
Hard membrane: Comparison between simulations and experiments from [24].

We consider three cases that we call, soft membrane, intermediate membrane, and hard membrane. We start with the case of the intermediate membrane (Fig 5a): the ‘normal’ case, which subsequently is compared to the soft (Fig 5b) and the hard membrane (Fig 6).

Fig 5a shows the comparison between simulations and experiments in the case of the intermediate membrane. The overall dynamics is very similar, but there are same noticeable differences. In the simulation, the maximum opening of the membrane is wider. This is due to the absence of the central chamber in the experimental set-up. Another minor difference occurs at the end of the cycle when the backpressure closes the valve completely. When closed, the experimental valve has a more elongated shape since the leaflets are longer. This is a consequence of the fact that, besides the main group N_R_, also Λ (eq 5) has a (minor) effect on the membrane. Experiments and simulation have the same N_R_, but not exactly the same Λ (see Table 1); some (minor) differences, therefore, are expected.

Fig 5b shows the comparison between simulations and experiments in the case of the soft membrane. The soft membrane can be considered defective since its position is completely reversed by the backflow. As in the previous case, there are some minor differences, but overall the membrane behaviour is well captured by the model. Similar deformation profiles have also been by other studies [15].

Fig 6 shows the comparison between simulations and experiments in the case of the hard membrane. Also the hard membrane can be considered defective since it does not completely opens. In this case, the comparison focuses on the membrane’s tip. At this location, the two leaflets slide one over the other and symmetry is lost. This phenomenon is captured in both the simulations and the experiments.

The loss of symmetry suggests that the simulations should account for the whole geometry and not only half of it (considering only one leaflet) [25–27].

Besides validating our model, this section also highlights the importance of N_R_. The values of Re, Λ and N_f_ between the simulations and in the experiments are different, but, since N_R_ is the same, the membrane behaviour in both cases is similar (with the little caveat about Λ as discussed above).

### Formation of solid aggregates

This section introduces solid aggregation in the DMHS. We consider two cases: solid deposits at the membrane surface, and formation of aggregates in the main flow. We generally indicate the first case as ‘calcification’ and the second as ‘clotting’. Our focus, however, is not to the formation and the evolution of actual calcifications and clots. These are very complicated biochemical phenomena [28] and their full dynamics is beyond the scope of this article. The goal is here to illustrate how, given a criterion for aggregation, this can be implemented in our model. Once this has been achieved, more complicated agglomeration models [10, 11] can be implemented.

**Fig 7:**
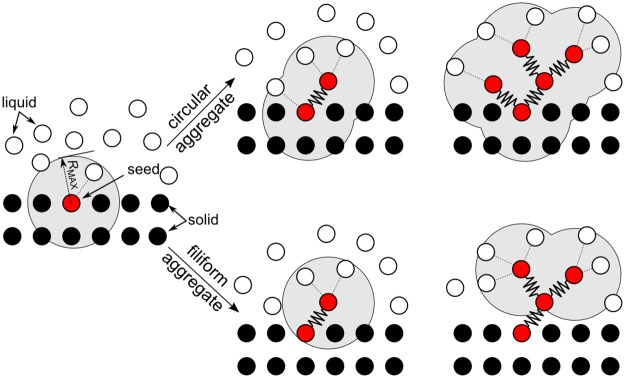
Algorithm for circular and filiform aggregates.

Both calcification and clotting imply the formation of solid aggregates developing from the liquid. In the DMHS framework, this can be achieved by changing the forces acting on certain particles from SPH to MSM. Fig 7 describes the algorithm used in the simulations. The procedure starts from an agglomeration seed. In our simulations, the seed is chosen arbitrarily, but it can depend on a specific criterion; for example, when local shear stress exceeds a threshold value, the particle at that location becomes a seed. Once the position of the seed is known, the algorithm propagates the agglomerate. Every *N* time-steps, it identifies all the particles within a distance *R*_MAX_ from the seed, and, with a certain probability, transforms some of the liquid-particles in solid agglomerate-particles by (i) changing the forces acting on the particles from SPH to MSM, (ii) and creating a bond between the seed and the newly created agglomerate-particle. The strength of the new bond determines the material properties of the agglomerate (see S3 Appendix). In our simulations, the probability of transforming a liquid-particle in an agglomerate-particle has been related to a fixed value (see S3 Appendix), but, as mention before, it can be associated to a specific criterion (e.g. shear stress threshold).

The algorithm repeats the above procedure iteratively to propagate the agglomerate further and new agglomerate particles create bonds only to other fluid particles and not to existing agglomerate particles. At the next time step, the previously generated agglomerate-particles become seeds; these seeds create new agglomerate-particles and so on as illustrated in Fig 7.

We can affect the final shape of the agglomerate by changing, as time progresses, the behaviour of the seeds. If the seeds are active all the time, they continue to create new agglomerate-particles around them until they are fully surrounded by the agglomerate. In this case, the overall shape of the agglomerate tends to be circular. If the seeds remain active only for one time step, the agglomerate propagates in one preferential direction and tends to assume a filiform (thread-like) shape (Fig 7).

**Fig 8:**
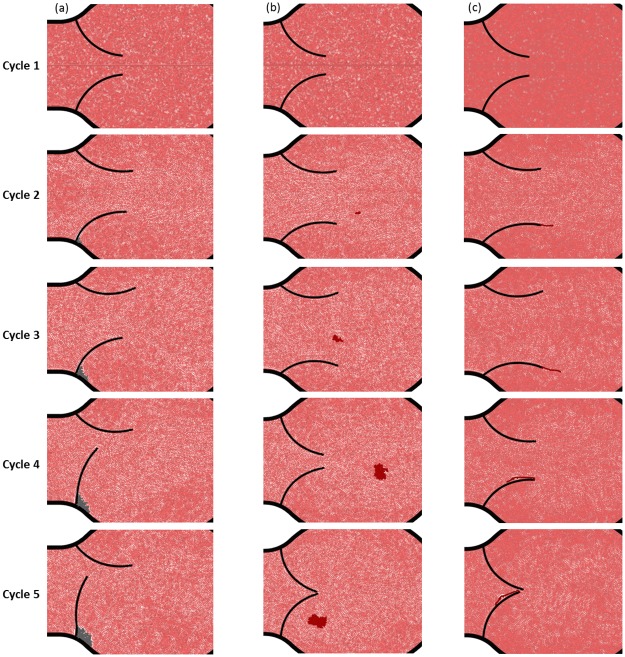
Solid aggregates: (a) ‘calcification’, (b) ‘free clot’ (c) and ‘filiform clot’.

Fig 8 shows three types of simulations where the algorithm is applied to three different configurations. The simulation parameters (*N*, *R*_MAX_, agglomeration probability, etc.) for all three cases are gathered in S3 Appendix. With the goal of obtaining a sizable aggregate in a few cycles, we accelerated the agglomerate formation by using higher aggregation probabilities. This is a typical technique used to study phenomena with very different timescales as those occurring in pipelines erosion.

In the first case (called ‘calcification’ in Fig 8a), the initial seed is located in the region between the membrane and the wall and the deposit propagates following the circular agglomeration algorithm illustrated in Fig 7. An interesting feature of the simulation is that, as time progresses, the agglomerate makes increasingly difficult the movement of the lower leaflet until it stops almost completely.

**Fig 9:**
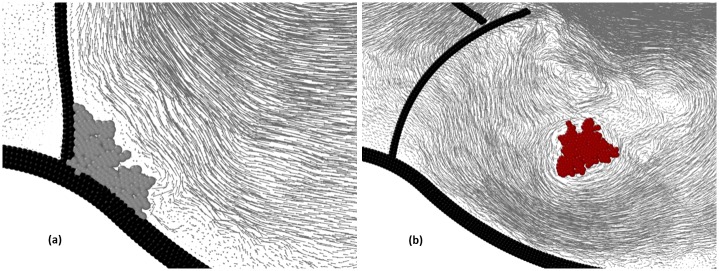
Velocity vectors illustrating how the presence of the aggregate affects the hydrodynamics: (a) ‘calcification’, (b) ‘free clot’.

In the second case (called ‘free clot’ in Fig 8b), the initial seed is located in the flow and the agglomerate propagates following the circular algorithm. The presence of a solid aggregate alters the hydrodynamics as indicated in Fig 9. Once a liquid particle transform into a solid particle, the fluid streamlines must change direction to account for the new solid-liquid boundaries. This feature would not be possible with mesh-based algorithms and highlights one of the advantages of discrete multi-physics.

The third case (called ‘filiform clot’ in Fig 8c) is similar to the previous case. This time, however, the initial seed is located at the tip of the leaflet and the agglomerate propagates according to the filiform algorithm. As the filiform aggregate grows, it moves alternately on the right and on the left of the membrane due to the oscillating flow.

**Fig 10:**
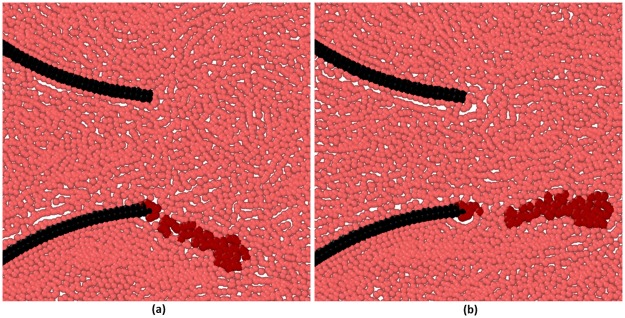
Fragmentation of the agglomerate.

We can emphasize another advantage of using a particle-based technique by introducing fragmentation. The drag between the fluid and the agglomerate creates internal stresses in the solid. These stresses tend to pull apart the agglomerate-particles that respond with a stronger binding force (according to equation J in S1 Appendix). At this point, we can slightly modify the algorithm and introduce a criterion for break-up: if the force between two agglomerate-particles exceeds a certain value, the bond breaks. In Fig 10, we used a threshold force of 1.3·10^−7^ N. At a certain point of the simulation, the threshold force is exceeded and the agglomerate breaks in two parts. One part remains attached to the leaflet; the other becomes free and moves unrestricted with the flow.

## Conclusions

Mesh-free methods are usually considered viable alternatives to traditional modelling, but have never enjoyed the same popularity of mesh-based techniques. Many mesh-free methods have been developed only in relatively recent years and offer, to the potential user, less available information, experience and software. On the other hand, a specific sub-set of mesh-free algorithm (e.g. SPH, DEM, CGMD, BD etc.) share a common particle-based framework that makes particularly easy their linkage in multi-physics problems. We call this approach *discrete multi-physics* and, in this paper, we show that, in certain circumstances, it is more than a mere alternative to traditional modelling. Discrete multi-physics can tackle, with relatively little effort, problems that are considered very challenging with mesh-based multi-physics. Elsewhere [14, 16, 21], we focused on solid-liquid flows where the dispersed phase is made of deformable, breakable, dissolving, melting or solidifying particles. Here, we apply the same approach to biological valves including the formation of solid aggregates in the flow and at the membrane surface. To the best of our knowledge, this is the first study to directly account for the hydrodynamics, the membrane deformation and the formation of solid aggregates at the same time and, as such, it has the potential to open a new prospective to the modelling of biological valves.

## Supporting information

S1 Appendix (DOCX)

S2 Appendix (DOCX)

S3 Appendix (DOCX)
