# Supplementary material for: Discrete multi-physics: A mesh-free model of blood flow in flexible biological valve including solid aggregate formation
Source: PLoS One. 2017 Apr 6;12(4):e0174795. doi: 10.1371/journal.pone.0174795 (PMC5383103; doi:10.1371/journal.pone.0174795)
Supplement: S1 Appendix — (DOCX) [file pone.0174795.s001.docx]

**S1 Appendix**

The DMHS version used in this study links SPH and CGMD. In this section, we provide a brief introduction to these techniques and how they are coupled together.

*1. Smoothed Particle Hydrodynamics (SPH)*

The SPH equations of motion are obtained from the discrete approximations of the Navier‑Stokes equation at a set of points, which can be thought as particles characterized by their own mass, velocity, pressure and density. The fundamental idea behind this approximation lies in the identity

$f(\text{r})=\iiint f(\text{r}')\delta\text{(r}-\text{r}')d\text{r}',$ (A)

where *f*(**r**) is a generic function defined over the volume V, the vector **r** is a three-dimensional point in V and *δ*(**r**) is the three-dimensional delta function. In the SPH formalism, the delta function is approximated by a smoothing Lucy kernel *W* with characteristic width *h* (smoothing length) such that

$\lim_{h\to0}W(\text{r},h)=\delta(\text{r}).$ (B)

This brings to the approximation

$f(\text{r})\approx\iiint f(\text{r}')W\text{(r}-\text{r}',h)d\text{r}',$ (C)

which can be discretised over a series of particles of mass *m* = *ρ*(**r**)*d***r** obtaining

$f(\text{r})\approx\sum_{i} \frac{m_{i}}{\rho_{i}}f(\text{r}_{i})W\text{(r}-\text{r}_{i},h),$ (D)

where *m_i_* and *ρ_i_* are the mass and density of the *i*^th^ particle, and *i* ranges over all particles within the smoothing Lucy kernel (i.e. |**r**−**r***_i_*| < *h*). Equation (D) represents the discrete approximation of a generic continuous ﬁeld and can be used to approximate the Navier‑Stokes equation

$m_{i}\frac{dv_{i}}{dt}=\sum_{j} m_{i}m_{j}(\frac{P_{i}}{\rho_{i}^{2}}+\frac{P_{j}}{\rho_{j}^{2}}+\Pi_{i,j})\nabla_{j}W_{i,j}+\text{f}_{i},$ (E)

where *v_i_* is the velocity of particle *i*, *W_i,j_* means *W*(**r_j_-r_i_**, *h*), ∇*_j_* denotes the gradient of the kernel with respect of the coordinate *r_j_*, *P* is the pressure, **f***_i_* a volumetric body force, and Π*_i,j_* introduces the viscous forces. Various expressions for the tensor Π*_i,j_* are available; here we use [1]

$\Pi_{i,j}=-\alpha h\frac{c}{\rho_{ij}}\frac{v_{ij}\text{r}_{ij}}{\text{r}_{ij}^{2}+bh^{2}},$ (F)

where *α* is a parameter (~1) used to ensure the stability of the simulation, *c* is the artificial sound speed in the liquid and *b* is a constant introduced to avoid singularities in the case of very close particles ($b$ ≈ 0.01). The value of *α* depends on the specific type of simulation; in this study we use α = 1 as done in previous studies [2]. We also tested different values of *h*, the one used (see S3 Appendix) is a compromise between accuracy and computational times. The smoothing length is slightly larger than the membrane thickness, and therefore, the fluid particles on one side of the membrane can “see” those on the other side. This avoids a partially filled kernel and has not noticeable consequences on the results.

In order to close equation E, an equation of state linking ρ and *P* is required. In this paper, we use Tait’s equation

$P(\rho)=\frac{c_{0}\rho_{0}}{7}\left[\left(\frac{\rho}{\rho_{0}}\right)^{7}-1\right],$ (G)

where *c*_0_ and ρ_0_ are, respectively the sound speed and density at zero applied stress.

*2. Coarse-Grained Molecular Dynamics (CGMD) / Mass-spring modelling (MSM)*

Molecular dynamics is a form of investigation where the motion and the interaction of a certain number of computational atoms or molecules are studied. In classical MD simulations atoms move according to the Newtonian equations of motion

$m_{i}\frac{dv_{i}}{dt}=-\frac{\partial}{\partial\text{r}}U_{tot}(\text{r}_{1},\text{r}_{2},...\text{r}_{N}),$ (H)

where *U_tot_* is the total interatomic potential, which can be divided into two main parts: non bonded and intramolecular. Non bonded forces are usually represented by the so-called Lennard-Jones potential, while the intramolecular forces are often divided in subgroups e.g.

$U_{\mathrm{int}ramolecular}=U_{bond}+U_{angle}.$ (I)

Each of these potentials can have different forms. In this study, we use the harmonic bond potential

$U_{bond}=k_{b}(r-r_{0})^{2},$ (J)

where *k_b_* a Hookean coefficient, *r*_0_ the equilibrium distance, and the harmonic angle potential

$U_{angle}=k_{a}(\theta-\theta_{0})^{2},$ (K)

where *k_a_* is an angular Hookean coefficient and θ_0_ the equilibrium angle,

Equations (I─K) are the basis for the ball-and-stick representation of molecules that can be coarse-grained to model macroscopic solids within the MSM framework. This approach can be employed to model macroscopic phenomena such as stretching and bending of solids under the effect of external forces. In the case under investigation, we divide the membrane in a certain number of notional particles and use the potentials of equations I and K to simulate its deformation. Fig 1 shows how bond and angle potentials are used in the membrane model. This component of the DMHS has been indicated sometimes as CGMD and sometimes as MSM. The mathematical formulation is the same: at small scales (e.g. microfluidic applications) the term CGMD is preferred [3], at larger scales the term MSM is preferred [4].

*3. Coupling the two models*

The interaction between the solid (CGMD particles) and the liquid (SPH particles) is defined by boundary conditions, which relate the behaviour of two adjacent materials at the common interface. There are three main types of phenomena that must be taken into consideration in designing these boundary conditions [5]: no-penetration, no-slip and continuity of stresses. In continuum mechanics, these conditions are often represented as

$(\frac{\partial}{\partial t}\text{u}-\text{v})\cdot\text{n}=\text{0 (no - penetration), }$ (L)

$(\frac{\partial}{\partial t}\text{u}-\text{v})\times\text{n}=\text{0 (no - slip),}$ (M)

and

$\sigma_{s}\text{n}=\sigma_{f}\text{(-n) (continuity of stress),}$ (N)

where **n** is the normal to the boundary, **u** the displacement of the solid, **v** the velocity of the liquid, σ*_s_* the stresses in the solid and σ*_f_* in the fluid.

In the particle framework, various no-penetration methods can be implemented [6] and an additional central force of the Lennard-Jones type is often used

$f(r)=K\left[\left(\frac{r^{*}}{r}\right)^{n_{1}}-\left(\frac{r^{*}}{r}\right)^{n_{2}}\right]\frac{\text{r}}{r^{2}},$ (O)

where *r** represents the repulsive radius of the particle, and *n_1_* and *n_2_* are usually set to 4 and 2, although also the original 12-6 Lennard-Jones values are sometimes used. The constant *K* is chosen on the basis of a characteristic velocity of the flow. The no-slip condition models the friction between the solid and the fluid. In finite-element numerical methods it is enforced by imposing that the two materials have the same velocity at the interface. In our particle framework, because we have particles of different nature (e.g. SPH and CGMD) we superimpose to each CGMD particle a SPH “ghost” particle. In this way, the particle is at the same time a CGMD and a SPH particle. When it interacts with a SPH particle from the fluid, it behaves as a SPH particle, but when it interacts with another CGMD particle, it behaves as a CGMD particle. The advantage of using a particle-particle representation is that, once both the no-penetration and no-slip boundary condition are enforced, the continuity of stress is automatically satisfied by the equation of motion (E).

**References**

1. Monaghan JJ, Gingold RA. Shock simulation by the particle method SPH. Journal of Computational Physics, 1983. 52(2): p. 374-389.

2. Shahriari S, Hassan IG, Kadem L. Modeling unsteady flow characteristics using smoothed particle hydrodynamics. Applied Mathematical Modelling, 2013. 37(3): p. 1431-1450.

3. Alexiadis A. A smoothed particle hydrodynamics and coarse-grained molecular dynamics hybrid technique for modelling elastic particles and breakable capsules under various flow conditions. International Journal for Numerical Methods in Engineering, 2014. 100(10): p. 713-719.

4. Alexiadis A, Stamatopoulos K, Wen W, Batchelor HK, Bakalis S, Barigou M, Simmons MJH. Using discrete multi-physics for detailed exploration of hydrodynamics in an in vitro colon system. Computers in Biology and Medicine, 2017, 81: 188-198.

5. Müller M, Schirm S, Teschner M, Heidelberger B, Gross M. Interaction of fluids with deformable solids. Computer Animation and Virtual Worlds, 2004. W 15: p. 159-171.

6. Ferrand M, Laurence DR, Rogers BD, Violeau D, Kassiotis C. Unified semi-analytical wall boundary conditions for inviscid, laminar or turbulent flows in the meshless SPH method. International Journal for Numerical Methods in Fluids, 2013. 71(4): p. 446-472.
