# Supplementary material for: Discrete multi-physics: A mesh-free model of blood flow in flexible biological valve including solid aggregate formation
Source: PLoS One. 2017 Apr 6;12(4):e0174795. doi: 10.1371/journal.pone.0174795 (PMC5383103; doi:10.1371/journal.pone.0174795)
Supplement: S2 Appendix — (DOCX) [file pone.0174795.s002.docx]

**S2 Appendix**

The mechanical properties of structures simulated with the DMHS depend on the constants *k*_a_ (equation J in S1 appendix) and *k*_b_ (equation K in S1 appendix) used in MD-like potentials. The mechanical properties of a real material, however, are measured by means of macroscopic properties such as the elastic modulus *E* [kg m^-1^ s^2^] or the flexural rigidity *F* [kg m^3^ s^-2^]. In order to compare the calculations with the experimental work, we need a relation to link *k*_A_ and *k*_B_ with *E* and *F*. In our case, since no torsional and shear stress are considered, we have [1]

$k_{a}=4\frac{EI}{L},$ (A)

and

$k_{b}=4\frac{EM}{L},$ (B)

where *E* is the elastic modulus, *I* = *wd^3^*/*12* the moment of inertia, *M* = *wd* the cross section, *w* the width of the membrane and *d* its thickness. In the particle framework, the membrane has an equivalent thickness *d* that, combining equation A and B, can be calculated by

$d=\sqrt{3\frac{k_{a}}{k_{b}}.}$ (C)

Using this value of *d*, we can calculate *I* and *M*, subsequently *E* from equation B, and finally *F* = *EI* from equation A.

**References**

1. Scarpa F, Adhikari S, Gil AJ, Remillat C. The bending of single layer graphene sheets: the lattice versus continuum approach. Nanotechnology, 2010. 21(12): p. 125702.
