# Supplementary material for: Discrete multi-physics: A mesh-free model of blood flow in flexible biological valve including solid aggregate formation
Source: PLoS One. 2017 Apr 6;12(4):e0174795. doi: 10.1371/journal.pone.0174795 (PMC5383103; doi:10.1371/journal.pone.0174795)
Supplement: S3 Appendix — (DOCX) [file pone.0174795.s003.docx]

**S3 Appendix**

There are two types of parameter required for the simulations: model parameters and simulation parameters. S3 Table list the numerical values used in the model parameters (simulation parameters are discussed in Results section).

**S3 Table. Model parameters used in the simulations.**

| SPH (Equations E−G in S1 appendix) | |
| --- | --- |
| **Parameter** | **Value** |
| Number of SPH wall particles (4 layers) | 3262 |
| Number of SPH valve particles (2 layers) | 272 |
| Number of SPH fluid particles | 49224 |
| Mass of each particles (fluid) | 6.6∙10^-5^ kg |
| Mass of each particles (wall and valve) | 12∙10^-5^ kg |
| Initial distance among particles Δ*r* | 2.5∙10^-4^ m |
| Smoothing length *h* | 6.25∙10^-4^ m |
| Artificial sound speed *c*_0_ | 27 m s^-1^ |
| Density *ρ*_0_ | 1056 kg m^-3^ |
| Time step *Δt* | (i) 10^-6^ s, (ii) 10^-7^ s |
| (i) Fix membrane, (ii) flexible membrane |  |
| CGMD (Equations J and K in S1 appendix) | |
| **Parameter** | **Value** |
| Angular coefficient *k_a_* | (a) 0.01 J, (b) 1 J, (c) 0.01 J |
| Hookian coefficient *k_b_* | (a) 3.1∙10^5^ J m^-2^, (b) 2.2∙10^6^ J m^-2^, (c) 9.8∙10^6^ J m^-2^ |
| Equilibrium distance *r*_0_ | 2.5∙10^-4^ m |
| Equilibrium angle *ϴ*_0_ | π/2 rad |
| BOUNDARIES (Equation O in S1 appendix) | |
| Constant *K* | 4∙10^-4^ J |
| Repulsive radius *r** | 2.5∙10^-4^ m |
| CLOT FORMATION (Section Formation of solid aggregates) | |
| Number of time step for clot formation *N* | 10^5^ s |
| *R*_max_ | (1) 2.28∙10^-4^ m, (2) 2.3∙10^-4^ m, (3) 2.5∙10^-4^ m |
| Agglomeration probability | 25 % |
| Max bonds per clot particle | (1) 6, (2) 6, (3) 2 |
| (a) soft, (b) intermediate, (c) hard | (1) calcification, (2) free clot, (3) filiform |
